# Empowering HIV-infected women in low-resource settings: A pilot study evaluating a patient-centered HIV prevention strategy for reproduction in Kisumu, Kenya

**DOI:** 10.1371/journal.pone.0212656

**Published:** 2019-03-06

**Authors:** Okeoma Mmeje, Betty Njoroge, Pauline Wekesa, Alfred Murage, Raphael O. Ondondo, Sheryl van der Poel, Mary A. Guzé, Starley B. Shade, Elizabeth A. Bukusi, Deborah Cohan, Craig R. Cohen

**Affiliations:** 1 Department of Obstetrics, Gynecology & Reproductive Sciences, University of California, San Francisco, San Francisco, CA, United States of America; 2 Kenya Medical Research Institute (KEMRI), Centre for Microbiology Research, Nairobi, Kenya; 3 Research Care and Treatment Program (RCTP)—Family AIDS Care & Education Services (FACES), Kisumu, Kenya; 4 Department of Obstetrics and Gynaecology, Aga Khan University, Nairobi, Kenya; 5 Masinde Muliro University of Science and Technology (MMUST), Kakamega, Kenya; 6 WHO/HRP (the UNDP/UNFPA/UNICEF/WHO/World Bank Special Programme of Research, Development and Research Training in Human Reproduction), Geneva, Switzerland; 7 Department of Medicine, Division of Prevention Services, University of California, San Francisco, San Francisco, CA, United States of America; Public Library of Science, UNITED KINGDOM

## Abstract

**Background:**

Female positive/male negative HIV-serodiscordant couples express a desire for children and may engage in condomless sex to become pregnant. Current guidelines recommend antiretroviral treatment in HIV-serodiscordant couples, yet HIV RNA viral suppression may not be routinely assessed or guaranteed and pre-exposure prophylaxis may not be readily available. Therefore, options for becoming pregnant while limiting HIV transmission should be offered and accessible to HIV-affected couples desiring children.

**Methods:**

A prospective pilot study of female positive/male negative HIV-serodiscordant couples desiring children was conducted to evaluate the acceptability, feasibility, and effectiveness of timed vaginal insemination. Eligible women were 18–34 years with regular menses. Prior to timed vaginal insemination, couples were observed for two months, and tested and treated for sexually transmitted infections. Timed vaginal insemination was performed for up to six menstrual cycles. A fertility evaluation and HIV RNA viral load assessment was offered to couples who did not become pregnant.

**Findings:**

Forty female positive/male negative HIV-serodiscordant couples were enrolled; 17 (42.5%) exited prior to timed vaginal insemination. Twenty-three couples (57.5%) were introduced to timed vaginal insemination; eight (34.8%) achieved pregnancy, and six live births resulted without a case of HIV transmission. Seven couples completed a fertility evaluation. Four women had no demonstrable tubal patency bilaterally; one male partner had decreased sperm motility. Five women had unilateral/bilateral tubal patency; and seven women had an HIV RNA viral load (≥ 400 copies/mL).

**Conclusion:**

Timed vaginal insemination is an acceptable, feasible, and effective method for attempting pregnancy. Given the desire for children and inadequate viral suppression, interventions to support safely becoming pregnant should be integrated into HIV prevention programs.

## Introduction

With the increased availability of antiretroviral (ARV) therapy, HIV-infected individuals are living with HIV as a chronic disease [1]. Those with stable infection are focused on achieving full integration into their communities and living productive lives, including having children [2–4]. In the early stages of the HIV epidemic, however, HIV-infected individuals were advised against having children, a decision that was fueled by fear and ethical concerns associated with the risk of HIV transmission in the setting of attempted pregnancy [3].

In sub-Saharan Africa (SSA), women disproportionately represent the majority of new HIV infections, which occur within stable sexual relationships [5, 6]. It is estimated that 44% of couples in Kenya are HIV-serodiscordant and that HIV transmission within serodiscordant partnerships may account for more than 60% of new HIV infections [7, 8]. HIV infection does not change an individual’s reproductive desires [1]. An estimated 20–50% of HIV-infected individuals still want to have children [7];. In SSA, comprehensive reproductive counseling and services that support safely becoming pregnant are currently limited for HIV-affected individuals and couples desiring children [1, 9]. The provision of reproductive services as harm-reduction techniques allow couples to achieve their reproductive goals while preventing HIV transmission to the uninfected sexual partner and developing fetus [10]. In female-positive/male-negative (♀+/♂-) HIV-serodiscordant couples, timed vaginal insemination (TVI) of semen during the fertile period of the menstrual cycle, as well as consistent male condom use that essentially eliminates the risk of sexual HIV transmission when attempting pregnancy are easy, safe, convenient, and inexpensive reproductive options [9, 11, 12]. Although current guidelines recommend ARVs in HIV-serodscordant partnerships through ARV initiation in the HIV-infected partner or pre-exposure prophylaxis (PrEP) in the uninfected partner, HIV RNA viral suppression is not guaranteed and may not be routinely assessed among patients on ARVs; and PrEP is not widely available or acceptable to uninfected partners [13–15]. Therefore, other methods of safely becoming pregnant for HIV-affected couples desiring children should be evaluated and integrated into comprehensive HIV prevention and reproductive health services [1, 16, 17].

We evaluated the acceptability, feasibility, and effectiveness of TVI as a safer method of becoming pregnant among ♀+/♂- HIV-serodiscordant couples in Kisumu, Kenya. We conducted a prospective pilot study in Kisumu, in the Nyanza region of Western Kenya, where the HIV prevalence is 18.7% [18]. Nationally, the HIV prevalence is higher among women (6.9%) than men (4.2%) [18]. The primary outcomes of this study were acceptability, feasibility, and effectiveness of TVI amongst ♀+/♂- HIV-serodiscordant couples planning a pregnancy in Kisumu, Kenya. The secondary outcomes were the incidence of sexual and/or vertical HIV transmission, adherence to the TVI procedures, the incidence of infertility in those who did not become pregnant with TVI, and prevalence of viremia among HIV-infected women unable to become pregnant after six cycles.

## Methods

### Study population and sampling

Study participants were recruited from six Family AIDS Care & Education Services (FACES)-supported and two other HIV care and treatment clinics in the Kisumu area. FACES provides care to 77,553 clients at 131 sites throughout Western Kenya as of December 2014 [19]. Eligible study participants were ♀+/♂- HIV-serodiscordant couples who self-reported the following: 1) monogamous relationship and disclosure of HIV status to the male sexual partner; 2) childbearing desires; 3) sexually active (at least three encounters per month) with expressed ability to consistently use male condoms; and 4) the women were aged 18–34 years. Couples were excluded if: 1) the woman was pregnant at the time of screening, enrollment, or during the observation period; 2) they self-reported sterilization or conditions associated with infertility in the couple; 3) they self-reported use of medications thought to be teratogenic at the time the study was designed (e.g., efavirenz); or 4) World Health Organization (WHO) clinical stage 3 or 4 disease.

Recruitment of eligible study participants occurred in four ways. First, study recruitment flyers were posted in the waiting rooms at the healthcare facilities in Dholuo and Kiswahili for interested HIV-infected individuals to initiate a call. Second, healthcare providers at the FACES-supported HIV care and treatment clinics were informed of the study and eligibility criteria during an informational session led by the Study Coordinator. The healthcare providers were instructed to refer eligible HIV-infected women to the Study Coordinator for further screening and possible enrollment. Third, the FACES HIV care and treatment clinic database was queried for HIV-infected women aged 18–34 who were clients at one of the six recruitment sites. The medical charts of these women were reviewed by the study staff to assess the HIV status of their most recent and/or documented partner, current method of contraception, prior pregnancies, and living children. Women fulfilling eligibility criteria at the time of chart review were identified and approached at their next clinic visit by study staff to discuss the study and their possible interest in participating. Finally, the most successful recruitment strategy employed the delivery of health talks focused on pregnancy planning, with emphasis on TVI, in the waiting room of the healthcare clinic. Any woman identified by any of the four recruitment strategies was instructed to discuss the study with her HIV-uninfected male partner and present with him as a couple for a study information session. After the information session, couples with a continued interest in study participation were scheduled for a screening visit to assess for possible underlying infertility in either partner, review the study procedures, and complete the medical chart abstraction of the HIV-infected female. ♀+/♂- HIV-serodiscordant couples fulfilling all eligibility criteria with an expressed interest in study participation and willingness to comply with the study procedures were offered study enrollment. Study participants were contacted by phone and visited at home in order to encourage continued study participation and decrease loss to follow-up.

### Data collection and validation

Questionnaires were developed and administered in English, Dholuo, or Kiswahili via audio computer-assisted self-interview (ACASI) software to assess the participants’ ease of use with the TVI procedures and adherence to the study protocol throughout the study period (S1 Appendix). Female participants also agreed to collection of clinical and demographic data at the time of study enrollment via medical chart review. These data were abstracted from their medical records using a study-specific data collection form. After enrollment, couples were observed for a minimum of two months to monitor the woman’s menstrual cycle and provide couples’ counseling and support to enhance consistent condom use. During the observation period, women were taught to identify their fertile period using the calendar method, along with assessment of cervical mucus consistency. The female partner presented on a monthly basis during the observation period with their menstrual calendars, which noted the days of menstrual bleeding and the nature of their vaginal secretions. The estimated mean menstrual cycle length was used to prospectively estimate the fertile window, approximately five days, for TVI for up to six menstrual cycles. The male partner presented at three months after enrollment or prior to initiation of the TVI procedures to complete the ACASI questionnaire and screening for sexually transmitted infections (STI). The study coordinator also conducted a routine monthly home visit to assess the couple’s well-being, emphasize condom use, and collect a self-administered vaginal swab, if indicated.

A self-administered vaginal swab was collected on a monthly basis at the time of a clinic or home visit outside of the estimated fertile period to assess the presence of prostate specific antigen (PSA), a surrogate marker of semen exposure. A positive PSA test noted on the ABAcard p30 (Abacus Diagnostics, West Hills, California) suggested condomless sexual intercourse within the prior 24 hours. A monthly urine pregnancy test (QuickVue One-Step hCG Urine Test, Quidel Corporation, San Diego, California, USA) was also performed. A rapid HIV test was performed by finger prick using Determine HIV rapid kit (Alere Medical Co. Ltd, Japan) every three months in the male partner and positive test results were confirmed by Unigold HIV rapid kit (Trinity Biotech PLC, Ireland) in accordance with the Kenyan National Guidelines for HIV voluntary counseling and testing [20].

Prior to TVI initiation, couples were screened for STIs (i.e. *Chlamydia trachomatis*, *Neisseria gonorrhoeae*, *Trichomonas vaginalis* (Gen-Probe Aptima assay by Gen-Probe Inc., San Diego, California, USA), *and Treponema pallidum* (Rapid Plasma Reagin, Becton-Dickinson, Baltimore, Maryland, USA; *T*. *pallidum* Haemagglutination Assay (TPHA, Randox Laboratory LTD, United Kingdom) and treated, if indicated. Women were also screened for bacterial vaginosis with microscopy and symptoms of STIs at their routine study visits–monthly in women and every three months in the male partner. A positive STI test resulted in empiric treatment of the couple. A test of cure was performed at least three weeks after treatment before continuation with the TVI procedures.

During the periovulatory period, couples were taught to collect semen using a water-based lubricated condom after timed sexual intercourse two days prior to ovulation, on the estimated day of ovulation, and two days after ovulation due to the estimation of ovulation, for up to six cycles or until pregnancy was noted. Used syringes were stained with methylene blue to detect the presence of vaginal epithelial cells. The presence of vaginal epithelial cells, which was used as an internal validation of TVI compliance [21].

A fertility evaluation was offered to couples who did not achieve pregnancy within six cycles—a semen analysis in the male after three days of sexual abstinence and a hysterosalpingo-contrast-sonography (HyCoSy) in the female to assess tubal patency and uterine and endometrial factors, and to quantify ovarian reserve (i.e. antral follicle count) in the early follicular phase [22]. Semen analysis was performed according to WHO standards [23]. All HyCoSy assessments to assess female fertility were performed by a single provider (AM), thus reducing potential inter-observer variance. The male partner was also screened for chlamydia, gonorrhea, HIV, trichomoniasis, and prior chlamydia infection with an anti-chlamydial antibody. HIV RNA viral load (COBA AmpliPrep/COBAS TaqMan® HIV-1 Test, v2.0 Roche Diagnostics, Mannheim, Germany) was also assessed in the HIV-infected female.

### Study measurements

For this analysis, demographic and clinical characteristics assessed from ACASI surveys conducted at study enrollment include: prior ARV use, history of STIs and pelvic inflammatory disease, treatment of STIs, number of prior pregnancies, number of living children, current method of contraception, frequency of condom use, and whether or not male partners were circumcised. Characteristics assessed at enrollment from the female’s medical chart review include: age, occupation, education, presence of an AIDS-defining illness, WHO disease stage, current ARV use, body mass index, CD4 count, and date of HIV diagnosis.

Acceptability and feasibility of the TVI procedures were assessed monthly in females and every three months in males using ACASI during the intervention phase with the following questions: “*How easy was it for you and your partner to follow the instructions for vaginal insemination and perform the procedure during the past month*,” on a scale of 1 to 6 (e.g. 1-very easy, 2-easy, 3-somewhat easy, 4-a little difficult, 5-very difficult, or 6-impossible) and “*In the past month*, *how would you describe how well you followed the instructions and procedures for the study*?” on a scale of 0 to 3 (0-poor, 1-fair, 2-well, or 3-very well). The effectiveness of TVI was assessed using incidence of pregnancy among female participants who initiated the TVI intervention. HIV transmission was assessed with rapid HIV tests among male participants and the HIV status of infants at nine months of age born to women who became pregnant during the TVI intervention phase, as self-reported by the mother. Adherence to the TVI procedures was measured by a positive test for vaginal epithelial cells on syringes used for the TVI procedures.

### Data analysis

Demographic and clinical characteristics of study participants, as well as incident pregnancy were described using frequencies, proportions, and medians when appropriate. Kaplan-Meier analyses were used to estimate time to pregnancy for all women who initiated the TVI intervention; women were censored from this analysis at the time they exited the study. We utilized repeated measures generalized linear models to predict means and assess change in the acceptability and feasibility measures over time during the TVI intervention, including terms to examine the interaction of pregnancy and time. Change over time was considered significant at an alpha level of 0.05. Birth outcomes and neonatal HIV status were self-reported by the HIV-infected female. Descriptive statistics were used to characterize the outcome of the fertility evaluations.

Data collected in ACASI were extracted to Microsoft Excel Version 2007 (Microsoft Inc., Redmond, Washington, USA). Excel was also used to collect and store baseline measures abstracted from female study participants’ charts. These data were imported into SAS Version 9.3 (SAS Institutes, Cary, North Carolina, USA) for further data management and analysis.

### Ethical considerations

Ethical approval was obtained from the Kenya Medical Research Institute (KEMRI) Ethical Review Committee and from the University of California, San Francisco, and the University of Michigan institutional review boards. The study procedures were in accordance with the ethical standards of the responsible committee on human experimentation (institutional and national) and the Helsinki Declaration of 1975, as revised in 2000. All study participants provided written informed consent in their preferred language (i.e. Kiswahili, Dholuo, or English). This study was registered on ClinicalTrials.gov (NCT01468753).

## Results

### Demographics

**Fig 1:**
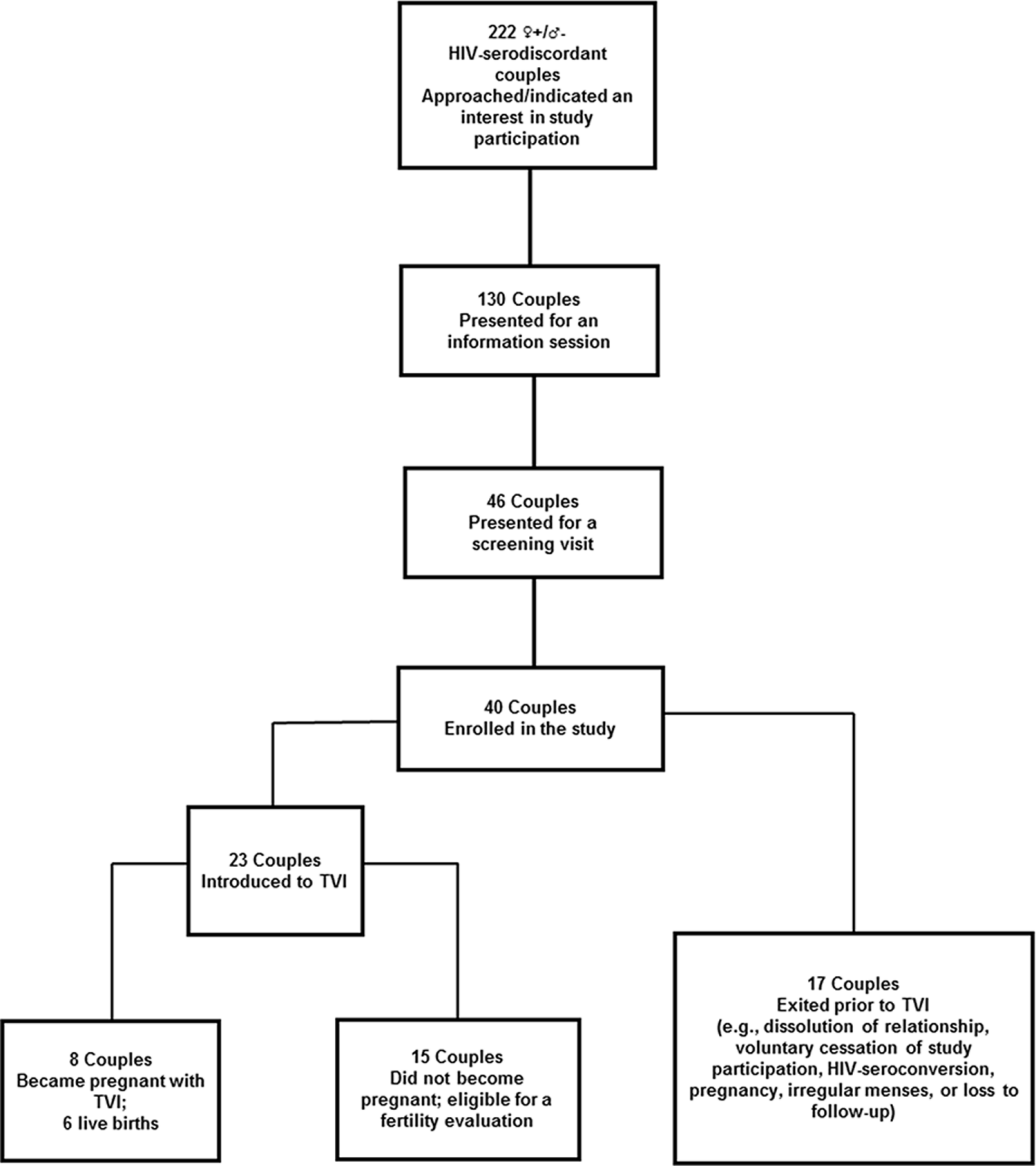
Study participants. ♀**+/**♂**-** Female positive/male negative.

Forty ♀+/♂- HIV-serodiscordant couples were enrolled in the study **(Fig 1)**.

Seventeen couples were exited for the following reasons: dissolution of the relationship (n = 4), voluntary cessation of study participation (n = 2), HIV seroconversion of the male partner (n = 2), irregular menses (n = 2), and loss to follow-up (n = 7). Sixty percent (n = 24) of women denied a prior history of STIs (*N*. *gonorrhoeae*, *C*. *trachomatis*, *T*. *vaginalis*, and herpes simplex virus infection). All women denied a history of pelvic inflammatory disease. One couple was diagnosed and treated for *T*. *vaginalis* infection prior to TVI. In another couple, the male partner was diagnosed with *T*. *pallidum* and treated according to Kenyan guidelines; they were later diagnosed and treated for *C*. *trachomatis* infection at an outside facility. After one cycle of TVI, the aforementioned couple was discontinued from the study due to their inability to comply with the study protocol and procedures. All couples had a negative test of cure prior to initiating the TVI procedure. Other sexually-associated infections that were diagnosed in our cohort included: bacterial vaginosis (n = 2), candida vaginitis (n = 1), and genital condylomas (n = 1).

The median age of women was 29 years (IQR 24–32). Approximately 53% (n = 21) of women were employed outside of the home, 35% (n = 14) completed secondary or post-secondary education, 62.5% (n = 25) were on ARVs at enrollment, and 72.5% (n = 29) self-reported consistent condom use at baseline **(Table 1)**. Twenty-three couples (57.5%) were introduced to TVI.

**Table 1:** Incidence and predictors of pregnancy at enrollment (n = 40).

Variables	Pregnant, n = 8 (%)	Non-pregnant, n = 32 (%)
**FEMALE PARTNER FACTORS**		
Age–Median (Q_1_ –Q_3_)	29 (22.0–32.0)	27 (23.5–31.0)
Work outside the home	3 (43)	18 (60)
Level of Education		
Some Primary	0 (0)	17 (71)
Some secondary	3 (43)	2 (8)
Some post-secondary	4 (57)	5 (21)
AIDS-Defining Illness		
Yes	0 (0)	4 (12)
No	7 (100)	28 (88)
WHO stage (initiation of care)		
1	5 (62)	19 (59)
2	3 (38)	8 (25)
3	0 (0)	3 (9)
4	0 (0)	2 (6)
Current ARV use	4 (50)	21 (66)
Prior ARV use^a^	4 (50)	21 (66)
History of STI (gonorrhea, chlamydia, trichomonas, HSV)	2 (25)	12 (38)
History of PID^a^		
Yes	0 (0)	0 (0)
No	8 (100)	32 (100)
BMI at enrollment–Median (Q1- Q3)	24.1 (22.2–26.8)	21.9 (19.1–24.6)
CD4 count/mm^3^ at enrollment median (Q1- Q3)	479 (447.5–739.5)	480.5 (367–574.5)
Number of prior pregnancies^a^		
0	1 (12)	5 (16)
1	2 (25)	9 (28)
2	3 (38)	12 (38)
3+	2 (25)	6 (19)
Number of living children^a^		
0	3 (38)	10 (31)
1	3 (38)	10 (31)
2	1 (12)	8 (25)
3+	1 (12)	4 (12)
Years since HIV diagnosis median (Q_1_ –Q_3_)	4.3 (1.3–7.4)	2.2 (1.0–5.5)
Current method of contraception		
None	0 (0)	11 (36)
Oral tablets	0 (0)	3 (10)
Depo Provera® (DMPA)	0 (0)	3 (10)
Other/traditional family planning	8 (100)	13 (43)
Condom use at enrollment		
100%	8 (100)	21 (66)
50–99%	0 (0)	8 (25)
<50%	0 (0)	3 (9)
**MALE PARTNER FACTORS**		
Age–Median (Q_1_-Q_3_)	33.5 (25.5–39.0)	33.5 (28.5–35.0)
History of STI^a^	2 (25)	10 (32)
Treated for STI^a^	2 (25)	7 (22)
Circumcised^a^	2 (25)	17 (53)
Number of living children^a^		
0	3 (38)	10 (31)
1	3 (38)	8 (25)
2	1 (12)	8 (25)
3+	1 (12)	6 (19)
Condom use at enrollment^a^		
100%	6 (75)	23 (72)
50–99%	2 (25)	7 (22)
<50%	0 (0)	2 (6)

ARV–antiretroviral therapy; WHO–World Health Organization; STI–sexually transmitted infection;

DMPA–Depot medroxyprogesterone acetate; HSV–herpes simplex virus; PID–pelvic inflammatory disease

^a^Indicates data was self-reported by patient

### Acceptability and feasibility of TVI

The estimated perceived mean ease of use during the first TVI cycle was described as “easy” or “somewhat easy” (2.96, 95% CI 2.4–3.5) by study participants. Ease of use did not differ by sex (p = 0.20), nor did it change over the course of the intervention by pregnancy status (p = 0.69). Amongst women introduced to TVI, 96.7% (n = 267 positive syringes per 276 cycle days) of their TVI syringes had evidence of vaginal insertion per staining of the syringes.

### Effectiveness of TVI

**Fig 2:**
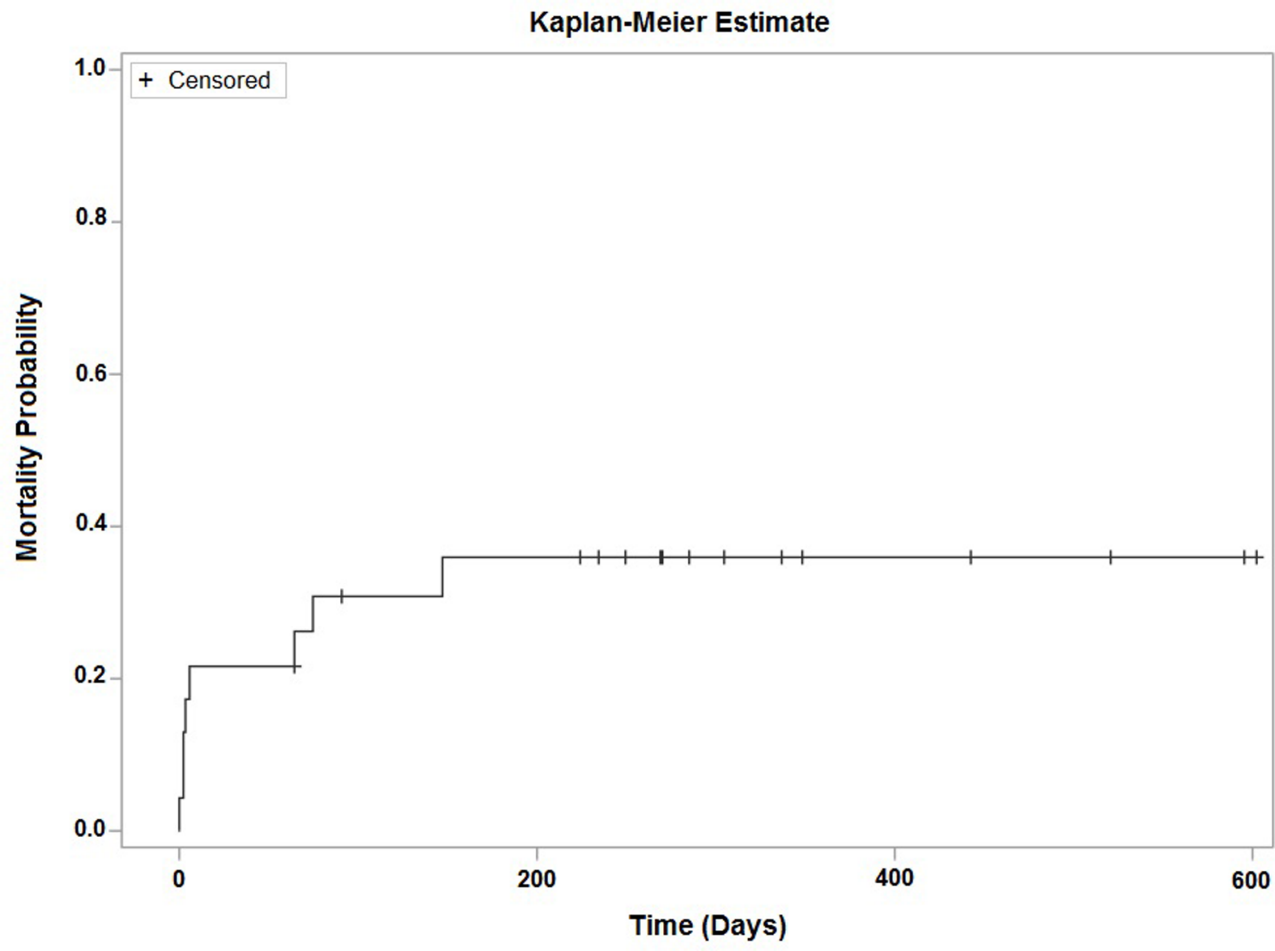
Kaplan-Meier plot–time to pregnancy (days) (n = 23^a^). ^a^Patients were only included in the analysis if an intervention start date was available. Follow-up time was calculated as time from intervention start date to conception date or intervention start date to study exit date, whichever occurred first.

Eight (34.8%) of the 23 women introduced to TVI became pregnant, which resulted in six live births without a case of HIV transmission to the neonate or partner. Two cases of neonatal demise occurred; one of a term (estimated gestational age of 39 weeks) neonate due to respiratory distress and the other due to complications of prematurity (estimated gestational age of 22 weeks). Accounting for loss to follow-up, our Kaplan-Meier analysis estimates that 36% (95% CI: 20–59%) of women introduced to TVI would become pregnant within 150 days, approximately five months, of TVI initiation (**Fig 2**).

### Non-pregnant couples

The 15 couples who did not become pregnant within six months were eligible for a fertility and HIV RNA viral load evaluation. Fourteen couples consented; seven couples completed both male and female fertility assessments; and 12 women completed viral load testing. Four women had bilateral fallopian tube occlusion, one male partner had decreased sperm motility, and five women had unilateral or bilateral fallopian tube patency. Seven (50%) women had an HIV RNA viral load (≥ 400 copies/mL) **(Table 2)**. The cost for a female and male fertility evaluation in Kenyan Shillings (KES) (1 USD ≈ 90 KES) was KES 13,300 (USD 147) and KES 8,880 (USD 99) respectively.

**Table 2:** Fertility evaluation findings.

Assessment	Result (n = 14)	Comment
**HyCoSy**	Bilateral/Unilateral tubal patency (n = 5)	Additional laparoscopic assessment and possibly in vitro fertilization in women with bilateral tubal occlusion
	Bilateral tubal occlusion (n = 4)	
	Inability to tolerate procedure (n = 2)	
	Withdrew consent for evaluation (n = 3)	
**AFC**	Range: 7–20	^**a**^All had reasonable ovarian reserve
**HIV RNA Viral Load** **(copies/mL)**	^b^Detectable in 7 women, range: 1,355–66,321	Consider ARV adherence and attempted pregnancy in the setting of a detectable HIV RNA viral load
**Other Pelvic Pathology**	Endometrial polyp(s) and fluid (n = 2)	Consider additional hysteroscopic assessment for endometrial pathology
	Submucosal fibroid in endometrial cavity (n = 1)	
	Unilateral dermoid cyst (n = 1)	
**Semen analysis** **(interpreted per WHO standards)**	Normal (n = 8)	Consider cultural and personal challenges to semen analysis
	^c^Borderline (n = 1)	
	Declined semen analysis (n = 5)	

HyCoSy–hysterosalpingo-contrast-sonography; AFC–antral follicle count

^**a**^Normal AFC >10 follicles on two ovaries combined

^b^All men were HIV-negative

^c^Suitable for intra-uterine insemination

## Discussion

### Acceptability, feasibility, and effectiveness of TVI

TVI is an acceptable, feasible, and effective method for ♀+/♂- HIV-serodiscordant couples attempting to become pregnant. The time to pregnancy achieved during this study mirrors that of HIV-unaffected couples who have no evidence of infertility [24, 25]. Study participants reported ease with the TVI procedures with support from clinical providers during the study period. In this pilot study, we employed the use of focused instructional aids for the TVI procedures, education and counseling sessions for couples eligible for TVI, and dedicated clinical staff to support couples using TVI as a reproductive method. Where appropriate, we recommend that clinical program directors implement these best practices to enhance the acceptability, feasibility, and effectiveness of TVI as a reproductive option for ♀+/♂- HIV-serodiscordant couples. Although we conducted our pilot study in Kisumu, Kenya, our findings are applicable to low-resource environments throughout the world and has high impact on empowering women in HIV-serodiscordant partnerships with an affordable and readily accessible reproductive option.

### Programmatic lessons from the field

Prior to implementation of TVI, programmatic costs should be considered due to STI screening and treatment, pregnancy and HIV testing, home visits and calls, and clinical staff time. In our cohort, all couples who became pregnant did so in one to three menstrual cycles. In order to decrease the distress associated with an inability to become pregnant and possible underlying infertility, healthcare providers should consider minimizing TVI attempts to no more than three menstrual cycles. As reproductive programs supporting pregnancy in HIV-serodiscordant couples are being developed and implemented, translation of research procedures should be considered. In the implementation phase, the use of PSA and syringe staining to assess consistent condom use may not be warranted.

All HIV-infected individuals in serodiscordant partnerships should be on ARVs in order to prevent HIV infection to the HIV-uninfected partner and unborn child. Furthermore, clinical support should be offered to HIV-affected couples planning a pregnancy to ensure HIV RNA viral suppression in the HIV-infected partner. We followed the existing national Kenya HIV treatment guidelines at the initiation of our study in 2012; however, these guidelines have changed, and all HIV-infected individuals in a HIV-serodiscordant partnership are currently eligible for ARVs regardless of CD4 count [26]. Although ARV initiation in the HIV-infected partner is protective against HIV transmission [27], couples should also be informed that HIV transmission may still occur [28, 29] due to poor ARV adherence, possible presence of HIV-resistant mutations [30], or condomless sex too soon after initiation of ARVs, which may limit the ability to achieve HIV viral suppression.

### Lessons following the fertility evaluations and recommendations

The findings of our fertility assessment highlight the need for providers to consider a fertility evaluation in couples preparing to attempt pregnancy prior to making recommendations on a safe reproductive method. Cost may be one of the significant barriers to a full fertility assessment; this should be addressed with pragmatic low-cost approaches that prioritize making a prompt definitive diagnosis and subsequently triaging couples to effective low-cost fertility interventions. Our fertility assessment was limited to diagnosis only and could only include referral for the provision of clinical services to address any identified fertility problem. Even though such information was informative, it underlines the agony of making a diagnosis and coming to an abrupt end-point as appropriate assisted reproduction techniques are either unavailable or currently unaffordable to most HIV-affected couples in low-resource settings. Strategies should also be considered to support and encourage male partners to complete their fertility evaluations, since a full couple’s evaluation is most meaningful in developing an appropriate plan of care for HIV-serodiscordant couples attempting pregnancy. Public health strategies, even in low-resource settings, must be put in place to address fertility interventions for couples in low socio-economic groups. A simplified fertility awareness questionnaire, such as FertiSTAT [31], may be used to assess for possible underlying fertility problems to reduce the risk of HIV exposure with unnecessary attempts at becoming pregnant. For those who are identified at higher risk for a fertility problem, through the use of a questionnaire, a clinical fertility assessment should be recommended and offered.

### Limitations

We encountered a few limitations stemming from a low uptake amongst those eligible for study participation and a 42% study drop-out rate. These limitations may be due to the novelty of TVI, requirement of disclosure of HIV status and male partner engagement, and sociocultural stigma associated with assisted reproduction.

### Future directions

There is an apparent research gap in the implementation and provision of safe reproductive options for HIV-serodiscordant couples desiring children. Our findings suggest that implementation studies are warranted to inform widespread integration of TVI into HIV care and treatment programs in low-resource settings. HIV-serodiscordant couples planning to have children should be supported in shared decision-making with a range of reproductive options that may include treatment as prevention, timed condomless intercourse, TVI, PrEP, and assisted reproductive technology services.

## Supporting information

S1 Appendix Clinical trial protocol. (DOCX)

S2 Appendix CONSORT checklist. (DOCX)

S3 Appendix De-identified study data. (ZIP)
