# Supplementary material for: Empowering HIV-infected women in low-resource settings: A pilot study evaluating a patient-centered HIV prevention strategy for reproduction in Kisumu, Kenya
Source: PLoS One. 2019 Mar 6;14(3):e0212656. doi: 10.1371/journal.pone.0212656 (PMC6402674; doi:10.1371/journal.pone.0212656)
Supplement: S1 Appendix — (DOCX) [file pone.0212656.s001.docx]

**TITLE OF THE PROJECT:**

**Pilot Study: Acceptability, Feasibility and Efficacy of Vaginal Insemination for Conception in Human Immunodeficiency Virus (HIV) Discordant Couples (female positive, male negative)**

**Desiring Pregnancy in Kisumu, Kenya**

**INVESTIGATORS AND INSTITUTIONAL AFFILIATIONS:**

**Principal Investigator:**

Okeoma Mmeje, MD, MPH

Clinical Fellow, Reproductive Infectious Disease

Department of Obstetrics, Gynecology and Reproductive Sciences

50 Beale Street, Suite 1200

San Francisco, CA 94105

[mmejeo@globalhealth.ucsf.edu](mailto:mmejeo@globalhealth.ucsf.edu)

(734) 972-4843

**Co-Principal Investigator**

Betty Njoroge, MB.ChB, MPH

Study Manager

Family AIDS Care and Education Services (FACES)

Centre for Microbiology Research (CMRI)

Kenya Medical Research Institute (KEMRI)

[betnjoroge@yahoo.com](mailto:betnjoroge@yahoo.com)

**Co-Investigators**:

Craig Cohen, MD, MPH

Professor

Department of Obstetrics, Gynecology and Reproductive Sciences

50 Beale Street, Suite 1200

San Francisco, CA 94105

[ccohen@globalhealth.ucsf.edu](mailto:ccohen@globalhealth.ucsf.edu)

(415) 597-9192

Elizabeth Anne Bukusi, MB.ChB, MPH, PhD, PGD

Chief Research Office and Deputy Director, Kenya Medical Research Institute (KEMRI)

Co-Director Research Care Training Program (RCTP)

Centre for Microbiology Research, KEMRI

[ebukusi@rctp.or.ke](mailto:ebukusi@rctp.or.ke)

Deborah Cohan, MD, MPH

Associate Clinical Professor

Department of Obstetrics, Gynecology and Reproductive Sciences

1001 Potrero Avenue, Ward 6D

San Francisco, CA 94110

[cohand@obgyn.ucsf.edu](mailto:cohand@obgyn.ucsf.edu)

(415) 206-3658

Maureen Adudans, MB.ChB, MPH

[maureenadudans@gmail.com](mailto:maureenadudans@gmail.com)

254 714 591 244

**ABSTRACT/SUMMARY:**

In sub-Saharan Africa, HIV is predominantly transmitted via discordant sexual relationships. With the availability of antiretroviral (ARV) medications, individuals infected with HIV can live relatively normal productive lives. Societal and cultural expectations as well as personal reproductive intentions drive HIV positive women in discordant relationships to conceive. Approximately 50% of HIV infected couples desire children. However, a safe and effective method of conception that minimizes the risk of sexual HIV transmission in HIV discordant couples with a positive woman and negative man (♀+/♂-) has yet to be examined. To date, published studies have evaluated assisted reproductive methods in HIV discordant couples with a positive man to decrease the risk of HIV transmission. We intend to evaluate the acceptability, feasibility and efficacy of vaginal insemination with semen for conception in (♀+/♂-) HIV discordant relationships in Kenya. In this pilot study, (♀+/♂-) HIV discordant couples desiring pregnancy will receive targeted reproductive counseling through the Safer and Healthy Conception Program (SHCP) for 6 months. This program will emphasize the consistent use of male condoms and teach couples assisted vaginal insemination for conception to minimize the risk of sexual HIV transmission. We will compare the frequency of male condom use before and after intervention with an audio computer-assisted self-interview (ACASI) validated by random measurement of prostate specific antigen (PSA) of vaginal secretions. The incidence of pregnancy following vaginal insemination will also be measured. We hypothesize that our findings will provide evidence to support the routine use of vaginal insemination as a safe method of conception in (♀+/♂-) HIV discordant couples. This pilot study is of significant public health importance because the use of vaginal insemination for conception in (♀+/♂-) HIV discordant couples is expected to reduce the likelihood of riskier sexual practices for childbearing and decrease the incidence of HIV in sub-Saharan Africa.

**INTRODUCTION/BACKGROUND:**

The HIV pandemic has taken the lives of reproductive-aged men and women globally.^1^ In resource-limited countries, the HIV epidemic has crippled the productivity and growth of nations for generations to come.^2^ With the increased availability of ARVs, people are living their lives with HIV infection as a chronic disease.^3^ Moreover, the risk of perinatal HIV transmission from a woman to her neonate is less than 2%, including resource-limited settings, if a woman is offered ARV therapy during pregnancy and throughout breastfeeding^4^. HIV discordance is defined as a stable relationship with a HIV positive partner and a HIV negative partner. In the early stages of the epidemic, medical organizations such as the Centers for Disease Control and Prevention (CDC), the American Board of Obstetrics and Gynecology (ACOG), and the American Society for Reproductive Medicine (ASRM) released statements discouraging assisted reproductive technology (ART) services in discordant HIV couples desiring conception. ^5^ This decision was fueled by fear and ethical concerns associated with the risk of HIV transmission in the setting of assisted reproduction.^5^ Therefore, the reproductive options and care of HIV discordant couples needs to be evaluated with the objective of allowing couples to fulfill personal reproductive goals while decreasing the risk of sexual HIV transmission.

In sub-Saharan Africa, women disproportionately represent the majority of new HIV infections which occur within stable sexual relationships.^6,7^ It is estimated that 44% of couples are HIV discordant in sub-Saharan Africa and that HIV transmission within discordant relationships may account for more than 60% of new HIV infections.^2,8^ A mathematical model estimates that the annual risk of HIV transmission from a woman to a man is 0.0022 and from a man to a woman is 0.0043 with an average of 100 acts of sexual intercourse per year.^9,10^ In low income countries, the risk of HIV infection per coital act in female-to-male and male-to-female transmission is 0.38% and 0.30% respectively.^11^ An estimated 20-50% of HIV positive individuals desire children.^8^ Being HIV positive modifies but does not remove reproductive desires.^1^ Comprehensive reproductive counseling and ART services are limited for HIV positive women desiring pregnancy in discordant relationships. In discordant relationships with a HIV-positive man, sperm washing with intrauterine insemination and in-vitro fertilization with intra-cytoplasmic sperm insemination (IVF-ICSI) have been effective at achieving pregnancy without any documented cases of HIV transmission using standard techniques.^12^ However, these techniques are not affordable for and available to discordant couples in which the woman is HIV positive in resource-limited settings. Furthermore, techniques utilizing sperm washing are relevant only for couples in which the HIV infected partner is male. HIV discordant couples in which the female is HIV infected do not require such laborious and expensive techniques to reduce the risk of HIV transmission during attempted conception.^12^ Vaginal insemination of semen during the fertile period is an easy and safe reproductive option, along with consistent male condom use can significantly reduce the risk of sexual HIV transmission for HIV positive women desiring pregnancy in a discordant relationship.^13,14,15^ Assisting HIV discordant couples in safer conception practices reduces HIV exposure and serves to minimize risk of HIV transmission.^16^

**JUSTIFICATION:**

The Kisumu Family AIDS Care and Education Services (FACES) clinic, the study site for this pilot study, participated in the Partners in Prevention Study, a randomized controlled trial of acyclovir for herpes simplex virus-2 (HSV-2) suppression to prevent HIV transmission.^17^ The FACES study team conducted a secondary analysis to characterize HIV discordant couples that conceived while enrolled in this trial.^18^ 532 HIV discordant couples were enrolled at the Kisumu, Kenya site, 61.7% of the infected partners were women: 189 (35.1%) of the women conceived during the study. Twenty (10.8%) seroconversions occurred among 186 HIV uninfected individuals in partnerships where pregnancy occurred compared to 21 (5.9%) seroconversions in 353 uninfected individuals in partnerships where conception did not occur resulting in a relative risk of 1.8 (95% CI 1.01-3.26). These data suggest that the intention to conceive among HIV discordant couples is contributing to the epidemic.^18^

To date all studies evaluating reproductive methods in HIV discordant couples have centered on interventions in which the male partner is HIV positive. There have been no studies examining the acceptability, feasibility, and efficacy of vaginal insemination in (♀+/♂-) HIV discordant couples. Thus, we plan to evaluate vaginal insemination with promotion of consistent male condom use with the intent of eliminating the risk of sexual HIV transmission and enhancing the repertoire of reproductive options for HIV positive women desiring pregnancy in discordant relationships.^16^

Societal and cultural expectations along with personal reproductive intentions drive HIV-positive women in discordant relationships to conceive.^20^ These expectations may result in risky sexual practices that contribute to the transmission of HIV. This is a public health issue in sub-Saharan Africa where HIV is endemic. Targeted reproductive healthcare services for (♀+/♂-) HIV discordant couples can reduce HIV transmission caused by risky sexual practices.^21^ The SHCP and the use of vaginal insemination will provide education, counseling and a reproductive option for HIV positive women in a discordant relationship desiring pregnancy.

**HYPOTHESIS:**

Specific Aim 1 (♀+/♂-) HIV discordant couples will accept vaginal insemination as a feasible option for conception as compared to natural conception.

Specific Aim 2 (♀+/♂-) HIV discordant couples enrolled in the SHCP will use male condoms consistently to significantly reduce the risk of HIV transmission to the uninfected partner.^14,19^

**GENERAL OBJECTIVES:**

To evaluate the acceptability, feasibility, and efficacy of vaginal insemination as a method of conception in (♀+/♂-) HIV discordant couples desiring pregnancy in Kenya.

**SPECIFIC OBJECTIVES:**

Specific Objective 1 (♀+/♂-) To evaluate the feasibility of vaginal insemination in HIV discordant couples as compared to natural conception.

Specific Objective 2 (♀+/♂-) To evaluate the consistent use of male condoms for the prevention of sexual (horizontal) transmission of HIV). ^14,19^

**DESIGN AND METHODOLOGY:**

Study Site:

The study will be conducted at the FACES affiliated HIV care and treatment clinics in Kisumu, Kenya in the Nyanza province of Western Kenya. These clinics provides HIV care and treatment for approximately 11,000 patients a year.

Study Population:

32 (♀+/♂-) HIV discordant couples will be enrolled in this pilot study. The inclusion criteria are the following: HIV discordant couple (♀+/♂-) desiring conception, monogamous relationship (minimum of three-month duration), disclosure of HIV status to the sexual partner, women 18-34 years of age, sexually active (at least three encounters per month), expressed ability to consistently use male condoms, and follow the study protocol with respect to study visits and use of vaginal insemination. The exclusion criteria are the following: pregnant (at the time of enrollment or run-in-period), women over 35 years of age (decreased fertility ≥ 35 years of age), self reported history of sterilization or infertility by either partner, use of teratogenic medication (e.g. Efavirenz), and clinical stage of HIV/AIDS 3 or 4.

Sampling:

Our sample size calculation is based on the frequency of self-reported male condom use in (♀+/♂-) HIV discordant couples. We estimate that HIV discordant (♀+/♂-) couples consistently use male condoms in 50% of sexual encounters with an average of nine sexual acts per month.^21,37^ We estimate the frequency of consistent male condom use after enrollment in the SHCP to be 95%. We will need to enroll 32 couples to detect a 45% difference in consistent male condom use after enrollment in the SHCP with 80% power and a two-sided alpha of 0.05. This sample size accounts for an anticipated 15% loss-to-follow-up rate and assumes an 80% intra-couple correlation in responses to frequency of consistent male condom use and sexual intercourse before and after study enrollment.

Procedures:

**Specific Objective 1: To measure and evaluate the acceptability, feasibility and efficacy of vaginal insemination for conception in** (♀+/♂-) **HIV discordant couples seeking childbearing in Kenya.** A pilot prospective cohort study will be conducted. Men and women will individually complete an ACASI assessing their knowledge, attitudes, and acceptability of vaginal insemination during the fertile period for conception at the time of study enrollment and after attaining pregnancy or completion of the study period, whichever comes first. Knowledge, attitudes, and acceptability will be measured using a validated 5-point Likert scale.

**Specific Objective 2:** **To measure the frequency of consistent male condom use.** In a pilot prospective cohort study, men and women will individually complete an ACASI assessing their frequency of sexual intercourse and male condom use at the time of study enrollment and after attaining pregnancy or completion of the study period, whichever comes first. A proxy for consistent male condom use will be the presence of PSA in vaginal secretions. PSA is the most commonly used and well established biomarker of semen exposure.^22,23,24^ All female subjects will collect a self-administered vaginal swab (SAVS) at a randomly selected time, excluding the fertile period, determined by study staff on a monthly basis to detect semen exposure.

Overview of Study Protocol:

Prior to screening and enrolling subjects in the study, we will conduct formative research that will provide community education and capacity building with community members and FACES healthcare providers. We will conduct two focus groups: HIV infected patients and their partners and healthcare providers. The intent of the focus groups will be to understand the patients’ and providers’ knowledge of safe reproductive methods for HIV discordant couples and perceptions of vaginal insemination. In addition, we intend to elicit from focus group participants the support services and needs of the patients and healthcare providers that will enable them to successfully enroll and remain in the study over the eight month period.

Thirty-two HIV positive women in discordant relationships desiring conception will be recruited from FACES over a two month period. Eligible couples will be enrolled in the study and followed for two months before introduction to the SHCP. During this run-in-period, partners will individually complete the ACASI assessing their frequency of sexual encounters and consistent male condom use. Women will be taught to follow their menstrual cycles and evaluate the consistency of their cervical mucus to determine the fertile period of their cycle. The determined cycle length and fertile period that was identified during the observation period will be used to prospectively determine the optimal days for vaginal insemination during the study. Throughout the study, women will collect a SAVS to detect the presence of PSA, a validated proxy of consistent male condom use, and have a urine pregnancy test performed. Men will have a rapid HIV screen collected every three months. The SHCP will teach women to identify ovulation, the menstrual cycle midpoint, by maintaining a calendar and assessing the consistency of cervical mucus.^25,26,27^ Consistent male condom use will also be emphasized.

Prior to introduction to the vaginal insemination procedures, couples will be screened for sexually transmitted infections (*Chlamydia trachomatis, Neisseria gonorrhoeae, Trichomonas vaginalis, and Treponema pallidum*) and treated if indicated. Nucleic amplification testing will be done on vaginal secretions for chlamydia and gonorrhea. Syphilis infection will be detected with non-treponemal antibody screening. Women will be questioned about the occurrence of any vaginal symptoms (pain, vaginal odor, change in vaginal discharge) during their monthly clinic visits. Men will be questioned about any STI related symptoms (pain, burning with urination, urethral discharge or the presence of lesions) at their study visit every 3 months. If the woman in the partnership is diagnosed with an STI, the male partner will be empirically treated for the infection. Similarly, if the man is diagnosed with an STI, the woman will be empirically treated for her presumed infection. A test of cure to document resolution of infection will be performed at least 3 weeks after treatment. The vaginal insemination procedures will continue after documented treatment of their infection.

On the insemination days, semen will be collected using a water based-lubricated male condom and then the semen will be drawn into a 10 cc (9.5 cm in length) needleless syringe. Women will then perform vaginal insemination with this collected semen within one hour of collection. They will be instructed to carefully place the syringe in the vagina until the base (hub) of the syringe reaches the introitus or opening of the vagina. Vaginal insemination will occur within one hour of collection 2 days prior to ovulation, on the day of ovulation and 2 days after ovulation for up to 6 months or until conception occurs. For example, if ovulation were on day 14 of a 28-day menstrual cycle, vaginal insemination will occur on days 12, 14 and 16 of the cycle. Women will be taught by the study staff how to successfully perform vaginal insemination after semen collection in their homes. There will be sample demonstration for all study participants after the run-in period following the established protocol (Appendix C). At the end of the demonstration, couples will be expected to practice and properly demonstrate the procedures for successful vaginal insemination. The vaginal insemination demonstration will be repeated on several occasions throughout the study period. At the end of the study or after a pregnancy is confirmed, an ACASI will be completed individually to assess the acceptability and feasibility of vaginal insemination. The protocols for SAVS and vaginal insemination will be performed by the female partner using validated techniques.^28,29^

Limitations

Our study has several limitations. A randomized control trial is not feasible given the ethical implications of withholding male condom use in a HIV discordant relationship. The proposed study will not have a control group. Couples will serve as internal controls with comparison of data obtained during the run-in-period and after enrollment in the SHCP. Vaginal insemination can reduce the risk of HIV transmission during attempted conception, but it may not be effective for couples in which the woman experiences anovulatory or irregular menstrual cycles.^30,31^ Furthermore, couples may not achieve a 95% frequency of consistent male condom use during the study however our intent is to evaluate the acceptability, feasibility and efficacy of vaginal insemination in (♀+/♂-) HIV discordant couples in a real-life clinical setting. Finally, the detection of vaginal epithelial cells on syringes used for insemination does not confirm it was properly used for insemination during the fertile period or that semen was deposited. The staining of syringes confirms vaginal placement but it does not confirm protocol compliance.^28,29^

Study Measurements

Each partner in the HIV discordant relationship will be a study participant. For the HIV positive woman, clinical information will be obtained from their FACES medical record using study specific data collection forms. Their baseline CD4 count, viral load, if noted, use of ARVs and date of diagnosis will be recorded. Demographic information for subjects will be collected at the time of study enrollment.

Outcome variables for specific aim 1 will be acceptability, feasibility and efficacy of vaginal insemination after protected timed intercourse. Acceptability and feasibility will be measured using a validated 5-point Likert scale administered with ACASI at the end of the study period. A questionnaire will be adapted from the validated South African Demographic and Health Survey of 1998 and the Concerns During Assisted Reproductive Technologies (CART) scale for the purpose of this pilot study.^32^ The use of ACASI has been shown to capture sensitive behaviors in high risk populations and has several advantages over face-to-face interview: standardization of data collection, useful in low literacy populations, and reduces social desirability bias.^33,34,35^

Outcome variables for specific aim 2 will be the frequency of consistent male condom use and sexual intercourse during the run-in-period compared to after exposure to the SHCP. Frequency of male condom use and sexual intercourse will be individually reported by the men and women with the ACASI. All women will randomly collect a SAVS on a monthly basis to detect semen exposure. PSA will be eluted from swabs and loaded into the sample well of the immuno-chromatographic strip test cassette according to the manufacturer’s instructions using the ABAcard® p30 (Abacus Diagnostics, West Hills, CA). Women will be considered to have had recent unprotected sexual intercourse (recent semen exposure) within the prior 24 hours if there are pink lines noted in the test and control areas of the ABAcard® p30. The lower limit of detection for the ABAcard® p30 is 4ng PSA/mL.^36^ The ABAcard® p30 rapid test is 100% sensitive (95% CI, 98-100%) and 96% specific (95% CI 93-97%) compared to the quantitative test in detecting >1.0ng PSA/mL vaginal swab eluate.^36^ Vaginal insemination will occur with a syringe. Used syringes will be placed individually in bags and returned to study staff two weeks after insemination. Staining of the syringe with methylene blue for vaginal epithelial cells will be a surrogate marker of vaginal insemination after ovulation, a validated technique used in microbicide trials.^28,29^ Vaginal epithelial cells have been noted up to four months on devices after vaginal insemination.^29^ The presence of vaginal epithelial cells after staining with methylene blue will be a surrogate for vaginal insemination. Conception will be measured by a urine pregnancy test two-weeks after estimated ovulation.

Prior to initiation of the vaginal insemination procedures, STI testing will be performed by DNA amplification. and will be repeated at further visits only if clinically indicated. Cervical swabs (female) and urine (male) will be collected at the visit prior to initiating vaginal insemination and will be tested for *N. gonorrhoeae*, *C. trachomatis*, and *T. vaginalis* using nucleic amplification tests (Gen-Probe Aptima assay by Gen probe Inc., San Diego, CA).

Serologic testing for *Treponema pallidum* will be performed using Rapid Plasma Reagin (RPR) (Becton-Dickinson, Baltimore, MD) for screening and confirmed using T. pallidum Haemagglutination Assay (TPHA, Randox Laboratory LTD, UK).

To establish eligible heterosexual HIV discordant partnerships, both the female and male partner will undergo rapid HIV testing at baseline to confirm HIV status. After enrollment, the HIV negative male partner will be tested every three months for HIV infection from enrollment through 6 weeks after documented pregnancy or after 6 months of vaginal insemination procedures. HIV serology testing will be determined by finger prick using Determine HIV rapid kit (Alere Medical Co. Ltd, Japan) and positives will be confirmed by Unigold HIV rapid kit (Trinity Biotech PLC, Ireland) in accordance to the Kenyan National Guidelines for HIV voluntary counseling and testing (VCT). On follow-up, HIV status for reactive HIV rapids will be confirmed by Vironostika HIV ELISA kit (BioMerieux Vironostika HIV Uni-Form II Antigen/Antibody ELISA, Marcy l’Etoile, France).

If the male partner were to seroconvert and become HIV positive during the course of the study, blood samples (approximately 21 mL/about 2 tablespoons) would be collected from the male and female partner for HIV genotyping. This analysis is intended to determine and/or confirm if the acquisition of HIV occurred in the recognized partnership by mapping of the viral genome. This test would be performed by the HIVR Lab located at KEMRI Kisian Centre in Kisumu, which is an affiliate of the KEMRI-UCSF-RTCP FACES lab. A CD4 cell count will also be assessed from the female and male. The results of the HIV genotyping will not be disclosed to the couple and will be used for study purposes only.

**ETHICAL CONSIDERATIONS**

Human Subjects and Informed Consent

(♀+/♂-) HIV discordant couples desiring pregnancy who fulfill eligibility criteria will be recruited from the FACES clinic. The couples enrolling in the study must demonstrate and agree to participate and comply with the study protocol for eight months. Study participants must be able to sign the consent form and speak English, Swahili or Dholuo (Luo). Proposal submissions and approval by the UCSF CHR and Kenya Medical Research Institute (KEMRI) ERC were complete as of September 2011. Comprehension of the study protocol, risks and benefits will be assessed by questioning and verbal recall in the subject’s primary language. If the study participant does not demonstrate an understanding of the study protocol, risks and benefits, the study protocol will be reviewed until the participant is able to demonstrate a thorough understanding. Each participant’s interest in childbearing will be confirmed at the time of their enrollment visit. Couples will complete the informed consent informational session and process together. However, they will independently complete the study consent form with the study staff in order to allow them to express any desires to decline study participation.

Participant Data:

HIV related clinical data will be collected from the medical chart of the HIV positive woman. The medical history and demographic information of the HIV-negative man will be obtained through participant interviews with study staff. All data relating to sexual behavior, frequency of male condom use, current and lifetime sexual partners and prior sexually transmitted infections will be collected with ACASI. No study specific data will be included in the HIV positive woman’s FACES medical chart, with the exception of a positive urine pregnancy test, if pregnancy results. The results of SAVS and syringe staining with methylene blue will be collected monthly during the study period and maintained in the project data files.

Potential Risks:

There is a risk of sexual HIV transmission to the HIV uninfected partner. In this study setting with a motivated cohort and emphasis on consistent male condom use, we estimate the risk of sexual HIV transmission is lower than the general population. Sexual HIV transmission may occur with unintended, unprotected sexual intercourse or with rupture of the condom. If rupture of the condom were to occur, study participants will be expected to report this event to the study staff. The exposure will be evaluated and post exposure prophylaxis (PEP) will be offered to the male partner for prevention of HIV acquisition. There is also a risk of vertical HIV transmission to the neonate during development and at the time of delivery, if pregnancy were to occur. Subjects may be at risk for psychological and emotional disturbance if sexual and/or vertical HIV transmission was to occur or pregnancy did not result following vaginal inseminations. We theorize that this is not specific to the study or greater in risk compared to HIV discordant couples (♀+/♂-) in the general population engaging in natural conception. Subjects may also feel discomfort with performing monthly SAVS and vaginal insemination.

Protection Against Risks:

The Institutional Review Boards at UCSF and KEMRI will review and approve the study protocol prior to initiation of the study. The Principle Investigator will ensure that all ethical research standards involving human subjects are met. To reduce the possibility of sexual HIV transmission, consistent male condom use is emphasized in the SHCP. Men will be tested for HIV every three months during the study. If they are found to be positive, they will be referred to care. During the screening visit, men will be asked if they are circumcised and if not, they will be counseled and offered referral for circumcision services. If pregnancy results following vaginal insemination, participants will be immediately referred to prenatal care after confirmation of pregnancy four weeks after the first positive urine pregnancy test and they will be counseled on the use of ARVs for the prevention of vertical HIV transmission, if they meet eligibility criteria established by the WHO. We will ensure a comfortable and safe environment that will allow subjects to discuss their feelings at study visits. The research staff will call subjects periodically to assess their well being and compliance with the study protocol. Women will be taught how to perform the SAVS and vaginal insemination to minimize possible discomfort. During the monthly clinic visit for women, they will be verbally screened for vaginal symptoms (pain, change in vaginal discharge, vaginal odor, or bleeding) and possible injury from the syringe with the vaginal insemination procedures. If indicated, they will be tested and treated for STIs, if necessary. Similarly, men will be questioned about any STI related symptoms (pain, burning with urination, urethral discharge or the presence of lesions) at their study visit every three months.

**DATA MANAGEMENT:**

All participants will be assigned a subject identification number (SID). All data entered into the study databases will be de-identified and only associated with a SID. We will maintain a double entry system for the data. Research records maintained on paper will be transferred to a web-based data management and storage system, Research Electronic Data Capture (REDCap®) and/or Microsoft Access by study staff. Electronic data obtained via ACASI will be stored on the UCSF server in password protected files. All paper research records will be kept in a password protected, locked filing cabinet located in a restricted-access room at the research center in Kisumu. Quality control of the SAVS for the detection of PSA and staining of syringes with methylene blue for vaginal epithelial cells will be performed by the Laboratory Manager. Electronic and paper study records including medical and personal identifiers will be stored in an access-limited room.

Statistical Analysis:

Specific Objective 1: To measure the acceptability and feasibility of vaginal insemination in (♀+/♂-) HIV discordant couples before and after enrollment in the SHCP, 5-point Likert scale items will be considered as interval-level data and analyzed using the Mann-Whitney test. The efficacy of vaginal insemination for conception will be assessed with the number of pregnancies and calculated as a person-time rate. The pregnancy rate will be adjusted by the number of vaginal inseminations in a Poisson regression.

Specific Objective 2: The frequency of consistent male condom use will be the change in reported percent of time male condoms are used during the run-in-period and eight months after study enrollment. We will test whether the within person change is greater than 0. With 32 couples an average within-person change in the frequency of consistent male condom use equal to or greater than 11% will be significant at the p=0.05 level. The analysis of consistent male condom use will be performed from the man’s perspective because he is at risk of HIV infection. The same analysis will be performed from the woman’s perspective. A kappa statistic will be computed to assess the agreement in the frequency of consistent male condom use between male and female partners in the relationship.

**TIME FRAME/DURATION OF THE PROJECT:**

| **Activity**  **Jan’12-Sept ‘12** | **Jan – March 2012** | **March – Sept**  **2012** | **Aug – Oct 2012** | **Oct - Dec 2012** |
| --- | --- | --- | --- | --- |
| Planning & Development   - IRB submission and approval at UCSF and Kenya Medical Research Institute (KEMRI) - Employ and Train Study Staff - Develop Study Protocols and data base |  |  |  |  |
| Recruitment and Enrollment of Subjects |  |  |  |  |
| Run in Period |  |  |  |  |
| Exposure to Intervention |  |  |  |  |
| Prospective study x 6 months |  |  |  |  |
| Data Analysis |  |  |  |  |
| Abstract and Manuscript Preparation |  |  |  |  |

**EXPECTED APPLICATION OF THE RESULTS:**

The data from specific aim 2 will help us characterize the frequency of consistent male condom use in (♀+/♂) HIV discordant couples desiring pregnancy in Kenya. Pregnancy rates along with the understanding of the acceptability, feasibility and efficacy of vaginal insemination will illustrate that this is a reproductive option for HIV-positive women in discordant relationships that desire pregnancy in resource limited environments. These findings will guide targeted reproductive healthcare services for (♀+/♂-) HIV discordant couples to reduce the risk of sexual HIV transmission.

**REFERENCES:**

1. D. Cooper, J. Harries, L. Myer, et al. "Life is still going on": Reproductive intentions among HIV-positive women and men in South Africa. Soc. Sci. Med. 2007; (65):274-283.

2. Sub-Saharan Africa. UNAIDS.2009. 2010 September 7.

3. M. Sauer,P. Chang. Establishing a clinical program for human immunodeficiency virus 1-seropositive men to father seronegative children by means of in vitro fertilization with intracytoplasmic injection.Am J Obstet Gynecol.2002; (186): 627-633.

4. R. L. Shapiro, M. D. Hughes, A. Ogwu, et al. Antiretroviral regimens in pregnancy and breast-feeding in Botswana N.Engl.J.Med. 2010; (362)24:2282-2294.

5. A. C. Thornton, F. Romanelli, J. D. Collins. Reproduction decision making for couples affected by HIV: A review of the literature. Top. HIV. Med. 2004;12: 261-67.

6. B. L. Guthrie, G. de Bruyn,C. Farquhar. HIV-1-discordant couples in sub-Saharan Africa: Explanations and implications for high rates of discordancy. Curr. HIV. Res. 2007; 54: 416-429.

7. T. C. Quinn,J. Overbaugh. HIV/AIDS in women: an expanding epidemic. Science. 2005; 308:1582-1583

8. L. T. Matthews, J. M. Baeten, C. Celum, et al. Periconception pre-exposure prophylaxis to prevent HIV transmission: benefits, risks, and challenges to implementation AIDS 2010; (24)13:1975-1982.

9. A. Vandermaelen,Y. Englert. Human immunodeficiency virus serodiscordant couples on highly active antiretroviral therapies with undetectable viral load: conception by unprotected sexual intercourse or by assisted reproduction techniques? Hum. Reprod. 2010; 252: 374-379.

10. D. P. Wilson, M. G. Law, A. E. Grulich, et al. Relation between HIV viral load and infectiousness: a model-based analysis. Lancet 2008; 372: 314-320.

11. M. C. Boily, R. F. Baggaley, L. Wang, et al. Heterosexual risk of HIV-1 infection per sexual act: systematic review and meta-analysis of observational studies. Lancet Infect. Dis. 2009; 92: 118-129

12. J. Ohl, M. Partisani, C. Wittemer, et al. Assisted reproduction techniques for HIV serodiscordant couples: 18 months of experience. Hum. Reprod. 2003; 186: 1244-1249.

13. P. Barreiro, A. Duerr, K. Beckerman, et al. Reproductive options for HIV-serodiscordant couples. AIDS. Rev. 2006; 83: 158-170.

14. A. M. Foss, M. Hossain, P. T. Vickerman, et al. A systematic review of published evidence on intervention impact on condom use in sub-Saharan Africa and Asia. Sex. Transm. Infect. 2007; 837: 510-516

15. L. Callegari, C. C. Harper, A. van der Straten, et al. Consistent condom use in married Zimbabwean women after a condom intervention. Sex. Transm. Dis. 2008; 356: 624-630.

16. L. T. Matthews,J. S. Mukherjee. Strategies for harm reduction among HIV-affected couples who want to conceive. AIDS. Behav. 2009; 13: Suppl 15-11.

17. C. Celum, A. Wald, J.R. Lingappa, A.S. Magaret, R.S. Wang, N. Mugo, A. Mujugira, J.M. Baten, J.I. Mullins, J.P. Hughes, E.A. Bukusi, C.R. Cohen, E. Katabira, A. Ronald, J. Kiari, C. Faraquhar, G.J. Stewart, J. Makhema, M. Essex, E. Were et al. Acyclovir and Transmission of HIV from Persons Infected with HIV-1 and HSV-2. N Engl J Med 2010; 36: 24-27

18. Sara G. Brubaker, Elizabeth A. Bukusi, Josephine Odoyo, et al. Pregnancy and HIV Transmission among HIV Discordant Couples in Clinical Trials in Kisumu, Kenya. HIV Medicine 2010; In Press.

19. R. Heffron, E. Were, C. Celum, et al. A Prospective Study of Contraceptive Use Among African Women in HIV-1 Serodiscordant Partnerships. Sex.Transm. Dis. 2010.

20. S. Gruskin, R. Firestone, S. Maccarthy, et al. HIV and pregnancy intentions: do services adequately respond to women's needs? Am. J. Public Health. 2008; (98)10:1746-1750.

21. M. Ezeanochie, B. Olagbuji, A. Ande, et al. Fertility preferences, condom use, and concerns among HIV-positive women in serodiscordant relationships in the era of antiretroviral therapy Int. J. Gynaecol. Obstet. 2009; 107: 297-98.

22. C. K. Mauck, G. F. Doncel, Biomarkers of Semen Exposure Clinical Working Group. Biomarkers of semen in the vagina: applications in clinical trials of contraception and prevention of sexually transmitted pathogens including HIV. Contraception 2007; 75: 6407-419.

23. L. Bahamondes, J. Diaz, N. M. Marchi, et al. Prostate-specific antigen in vaginal fluid after exposure to known amounts of semen and after condom use: comparison of self-collected and nurse-collected samples. Hum. Reprod. 2008; (23)11: 2444-2451.

24. M. Macaluso, L. Lawson, R. Akers, et al. Prostate-specific antigen in vaginal fluid as a biologic marker of condom failure. Contraception. 1999; 593:195-201.

25. C. D. Lynch, L. W. Jackson,G. M. Buck Louis. Estimation of the day-specific probabilities of conception: current state of the knowledge and the relevance for epidemiological research. Paediatr. Perinat. Epidemiol. 2006; 20 Suppl 13-12.

26. M. J. Zinaman. Using cervical mucus and other easily observed biomarkers to identify ovulation in prospective pregnancy trials. Paediatr. Perinat. Epidemiol. 2006; 20 Suppl 126-29 .

27. B. Scarpa, D. B. Dunson,E. Giacchi. Bayesian selection of optimal rules for timing intercourse to conceive by using calendar and mucus. Fertil. Steril. 2007; 884: 915-924.

28. A. Wallace, M. Thorn, R. A. Maguire, et al. Assay for establishing whether microbicide applicators have been exposed to the vagina. Sex. Transm. Dis. 2004; 318: 465-468.

29. A. R. Wallace, A. Teitelbaum, L. Wan, et al. Determining the feasibility of utilizing the microbicide applicator compliance assay for use in clinical trials. Contraception. 2007; 761: 53-56.

30. Harlow, Sioban D., Schuman, Paula, Cohen, Mardge. Effect of HIV Infection on Menstrual Cycle Length JAIDS. 2000; 24: 68-75.

31. Clark, Rebecca A., Mulligan, Kathleen, Stamenovic, Eva et al. Frequency of Anovulation and Early Menopause in Selected Adult AIDS Clinical Trials Group Studies. The Journal of Infectious Disease. 2001; 184: 1325-1327.

32. Department of Health, Medical Research Council and Macro International Incorporated. South Africa Demographic and Health Survey 1998, Full Report. 2002; http://www.doh.gov.za/facts/1998/index.html. Accessed - 2010September 18.

33. E. Pluhar, M. McDonnell Holstad, K. A. Yeager, et al. Implementation of audio computer-assisted interviewing software in HIV/AIDS research. J. Assoc. Nurses AIDS Care. 2007; 184:51-63.

34. A. M. Minnis, A. Muchini, S. Shiboski, et al. Audio computer-assisted self-interviewing in reproductive health research: reliability assessment among women in Harare, Zimbabwe. Contraception. 2007; 75: 159-65.

35. E. M. van der Elst, H. S. Okuku, P. Nakamya, et al. Is audio computer-assisted self-interview (ACASI) useful in risk behaviour assessment of female and male sex workers, Mombasa, Kenya? PLoS One. 2009; 45: e5340.

36. M. M. Hobbs, M. J. Steiner, K. D. Rich, et al. Good performance of rapid prostate-specific antigen test for detection of semen exposure in women: implications for qualitative research. Sex.Transm. Dis. 2009; 368: 501-506.

37. R. H. Gray, M. J. Wawer, R. Brookmeyer, et al. Probability of HIV-1 transmission per coital act in monogamous, heterosexual, HIV-1-discordant couples in Rakai, Uganda. Lancet. 2001; 357:1149-1153.

**Appendices**

Appendix A: Role of Investigators

Appendix B: Study Flow Diagram

Appendix C: Vaginal Insemination Procedures

**Appendix B:**

**OVERVIEW OF STUDY PROTOCOL**

(♀+/♂-) HIV discordant couples desiring conception receiving care in FACES

Run in Time: 2 months

- Baseline ACASI
- Random vaginal swabs for PSA (each month)
- Monitoring of ovulation cycles
- HIV surveillance
- HIV Antibody screen (Male)

Safer and Healthy Conception Program

Monthly Evaluations x 6 months

**Male Partner:**

-HIV Antibody screen every 6 weeks

**Female Partner:**

-Random vaginal swab for PSA (every month)

-Vaginal insemination every other day around ovulation monthly

-Monthly pregnancy test

Post-Intervention ACASI

**APPENDIX C**

**Vaginal Insemination Procedures**

1. Two days before ovulation, engage in sexual intercourse within 24 hours with a water based -lubricated male condom.

**WATER BASED LUBRICATED MALE CONDOM**


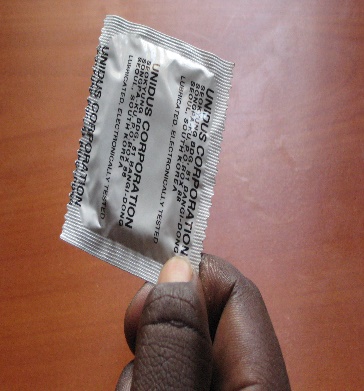

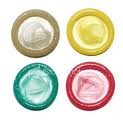


1. After ejaculation, collect semen from the male condom in a 10 cc syringe like the diagram below. Place the syringe tip just below the semen level in the male condom and pull back on the plunger. If there are air bubbles, hold the syringe with the tip up, and tap the syringe to make the bubbles rise. Push the air bubbles out while being careful not to spill any semen. If there is still more semen, pull back on the plunger again and tap the bubbles and push out the air again if needed.

**STEPS FOR SEMEN COLLECTION**


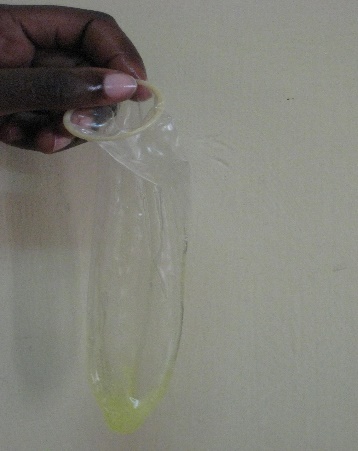

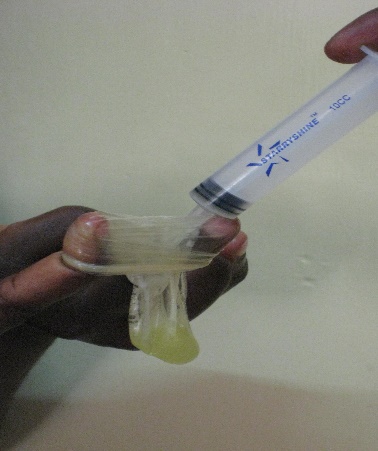


**SYRINGE FILLED WITH SEMEN**


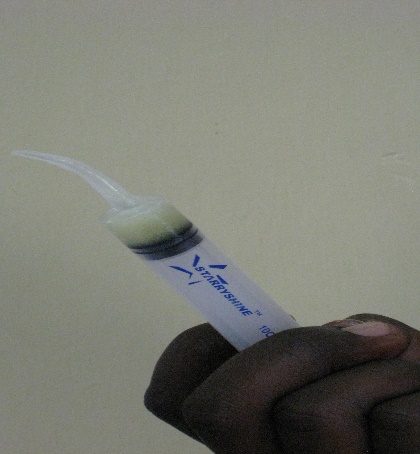


1. **Insert** syringe in vagina till the base of the syringe is at the opening of the vagina and gently deposit 1-2 cc of semen within one hour of collection.
2. Lay flat for one hour with hips elevated.
3. Repeat these procedures on the day of ovulation and two days after ovulation.

****You will perform insemination three times during the fertile period (2 days before ovulation, on ovulation and 2 days after ovulation).****
